# Upregulation of Serum miR-132 and miR-152 in Vietnamese Patients with Heart Failure

**DOI:** 10.21315/mjms2024.31.4.7

**Published:** 2024-08-27

**Authors:** Diem My Vu, Anh Phuong Huynh, Nhu Nhat Quynh Nguyen, Niem Van Thanh Vo, An Bac Luong, Thanh Cong Nguyen, Anh Vu Hoang

**Affiliations:** 1 Center for Molecular Biomedicine, University of Medicine and Pharmacy at Ho Chi Minh City, Ho Chi Minh City, Vietnam; 2 Cardiovascular Center, University Medical Center, Ho Chi Minh City, Vietnam

**Keywords:** serum miR-132, serum miR-152, heart failure biomarker

## Abstract

**Background:**

MicroRNAs (miRs) are emerging targets for the diagnosis, prognosis and treatment of heart failure (HF). Accumulated evidence showed that microRNA-132 (miR-132) and microRNA-152 (miR-152) play critical roles in the development of multiple pathological processes of the heart. Although their upregulations have been detected in the failing hearts of humans and animal models, little is known about the circulating levels of miR-132 and miR-152 in patients with HF.

**Methods:**

Our study was conducted from January 2022 to August 2022 at the Cardiology Department of the University Medical Center in Ho Chi Minh City, Vietnam. During study period, 36 participants were consecutively enrolled, including 18 HF patients and 18 patients who age and sex matched the non-HF controls. Serum samples of study participants were collected on admission and the expression levels of miR-132 and miR-152 were measured by quantitative reverse transcription polymerase chain reaction (RT-PCR). The comparative cycle threshold method (ΔCt) was applied to calculate the relative expression of miRs.

**Results:**

The miR concentration in HF group was significantly lower than that in the control group. In contrast, the serum levels of miR-132 and miR-152 were significantly higher in HF patients. Further analyses of receiver operating characteristic (ROC) curve showed that miR-132 and miR-152 individually had moderate diagnostic potential for HF (with area under curve [AUC] values of 0.713 and 0.698, respectively). A positive correlation between these miRs was also confirmed.

**Conclusion:**

Serum miR-132 and miR-152 were upregulated in Vietnamese patients with HF and may serve as candidate biomarkers for diagnostic purposes.

## Introduction

Heart failure (HF) is a complex and multifactorial syndrome induced by abnormal heart structure or function that results in insufficient cardiac output (1, 2). It has become a major global health issue, affecting over 64 million people worldwide (3). Despite remarkable improvements in drug development and therapeutics, HF hospitalisation and fatality rates remain high (4). Data analyses from approximately 1.5 million HF cases reported a 5-year mortality for 50%–75% of patients in particular populations (5, 6). Moreover, its prevalence has been predicted to increase by 46% between 2012 and 2030 due to rising numbers of the aging population and people affected by comorbidity diseases, such as obesity, hypertension and diabetes (7). Therefore, the development of novel diagnostic tools and therapeutic approaches is urgently needed to improve patient care.

MicroRNAs (miRs) are a class of endogenous, small, non-coding RNAs (∼21–25 nucleotides) that simultaneously regulate multiple gene expressions at the post-transcriptional level by pairing with the target mRNAs (8–10). In recent years, cumulative evidence has demonstrated that miRs play a pivotal role in the normal development and functioning of the heart (10). The dysregulation of miRs has been associated with various heart pathological processes such as cardiac remodeling, hypertrophy and apoptosis (11). Certain miRs have been reported to specifically increase or decrease in a failing human heart (9, 12, 13). Moreover, since they can be detected in blood, significant research has been conducted to investigate circulating miRs as potential biomarkers for different cardiovascular diseases such as coronary artery disease (14), dilated cardiomyopathy (15) and HF (16, 17).

Among the miRs associated with HF, microRNA-132 (miR-132) has been described as a cardiac-abundant miR that plays a major role in the regulation of myocardial hypertrophy and contractile function (18, 19). It has been reported to target several key factors in HF, such as the anti-hypertrophic transcriptional factor Forkhead Box O3 (FoxO3) (19), the sarcoplasmic/endoplasmic reticulum Ca^2+^-ATPase (SERC2a) in heart contractility (20) and the Ras GTPase-activating protein 1 (RASA1) of angiogenesis (21). MiR-132 has been further connected with HF through its interaction with the arginine vasopressin hormone, which regulates body fluid retention and cardiovascular contractibility (22, 23). Increased level of miR-132 was observed in the heart of human and animal models of HF (19, 20, 24). Remarkably, the clinical trial of miR-132 antisense drug has resulted in improved cardiac functioning of HF patients (25).

MicroRNA-152 (miR-152) is a member of the miR-148/152 family and is a highly conserved miRNA involved in cell proliferation, differentiation and survival (26). In the heart scenario, an interaction between miR-152 and DNA methyltransferase 1 (DNMT1) has been shown to induce cardiac fibrosis and result in HF (27). In addition, miR-152 negatively regulates the low-density lipoprotein receptor-related protein 6 (LRP6) receptor to mediate myocardial infarction (28). miR-152 upregulation is associated with structural remodeling of the heart through mitochondrial homeostasis alteration (29). In preclinical investigation, cardiac-specific overexpression of miR-152 resulted in the rapid progression of HF, whereas its inhibition exerted protective effects (30). The highest level of miR-152 was confirmed in an analysis of around 500 miRs from cardiac tissue of end-stage HF patients (30).

Although the biological importance and therapeutic potential of miR-132 and miR-152 have been demonstrated in different preclinical and clinical studies of HF, data on their circulating levels remain scarce. Since they may serve as useful indicators for HF and treatment response, further validation is recommended to support the translational application of circulating miR-132 and miR-152. In addition, it is suggested that ethnic disparity may have an impact on the circulating miRNA profiles of a particular population. Thus far, circulating miRs associated with HF have not been reported in our population. In this work, we analysed the expression levels of serum miR-132 and miR-152 in Vietnamese patients with HF and assessed whether they could serve as candidate biomarkers for HF diagnosis.

## Methods

### Study Design and Population

A cross-sectional study was conducted at the Cardiology Department of the University Medical Center, Ho Chi Minh City, Vietnam, from January 2022 to August 2022. Study participants were selected by reviewing medical records and convenience sampling was employed. The inclusion criteria for the HF group were as follows: ≥ 18 years old; a confirmed diagnosis of stable HF for more than 3 months, according to the 2016 Guidelines of the European Society of Cardiology (31), implementation of the HF management protocol issued by the Ministry of Health of Vietnam in 2020; and a New York Heart Association Classification of II–IV, irrespective of the cause and the left ventricular ejection fraction. The exclusion criteria for all participants were: acute myocardial infarction within a month before the study period, autoimmune disease, known malignancy, renal failure, surgery or transplantation within 3 months before the study period, pregnancy and breastfeeding. The controls were matched to the HF cases by age (± 5 years old) and gender. A total of 18 HF patients and 18 non-HF controls were included in this study.

### Sample Collection and Handling

A peripheral venous blood sample was collected from each participant in a serum collection tube, left to stand for 30 min at room temperature (RT) and then centrifuged at 4,400 rpm for 15 min at RT. After centrifugation, the serum was transferred into a new RNase/DNase-free tube and stored at −70 °C for subsequent experiments.

### Isolation of miRNAs

Total miR was extracted by using a Hybrid-R^TM^ miR extraction kit (#325-150, GeneAll Biotechnology, Korea). Briefly, 200 μL of serum was mixed with 500 μL of RiboEX^TM^ and incubated for 5 min at RT. Then, 200 μL of chloroform was added; and the mixture was vigorously shaken for 15 s, incubated for 2 min at RT and centrifuged at 12,000 g for 15 min at 4 °C. The mixture was transferred to a column type B and centrifuged at ≥ 10,000 g for 30 s at RT. The pass-through solution was collected and mixed with one volume of 100% ethanol, transferred into a type W column and centrifuged at ≥ 10,000 g for 30 s at RT. The column was washed twice with 500 μL RBW and once with 500 μL RNW via centrifugation at ≥ 10,000 g for 30 s at RT. In a new RNase/DNase-free tube, 25 uL of RNase-free water was added to the column before centrifugation at ≥ 10,000 g for 1 min at RT. Purified miR was stored at −70 °C for further experiments. miRNA concentration was determined using a NanoDrop 2000 spectrophotometer (NanoDrop Technologies, USA).

### miRNA Quantification

Reverse transcription was conducted using a miRCURY LNA RT Kit (#339340, Qiagen, Germany). The reverse transcription reaction was then diluted with water (1:5 ratio) and miR expression was measured using a miRCURY LNA SYBR Green PCR Kit (#339345, Qiagen, Germany) with pre-designed miRCURY LNA miR PCR assays for miR-132 and miR-152 (#339306). miR-103a was used as the reference as previously described (32). The primer sequences used for miR detection are listed in Table 1. The PCR was performed by exposing the reaction mixture to 95 °C for 2 min and then to 95 °C for 10 s and 60 °C for 30 s for 45 cycles. The comparative cycle threshold method (ΔCt) was applied to calculate the relative expression levels of the miRs and a Ct value ≥ 40 was considered undetermined. All samples were tested in duplicate.

### Statistical Analysis

Continuous variables are presented as mean (SD) unless otherwise stated. Categorical variables are presented as numbers and percentages. The normality of distribution was assessed using the Shapiro-Wilk test. Continuous variables were compared using a *t*-test unless otherwise noted. Pearson’s chi-squared test was used for the comparison of categorical variables unless otherwise stated. The area under the receiver-operating characteristic (ROC) curve was computed to assess the diagnostic potential of each miR. The optimal diagnostic points for each of the miRs were assessed at cutoff values using the largest Youden’s index. A *P*-value of < 0.05 was considered statistically significant. Data were analysed using SPSS version 25.0 (IBM, USA) and GraphPad Prism 8 (GraphPad Software, Inc., USA).

## Results

### Characteristics of the Participants

The baseline characteristics of all study participants are shown in Table 2. The recruitment age range was 22 years old–75 years old for the HF group and 27 years old–79 years old for the control group. The participants’ mean age, clinical comorbidities and medications were comparable between the HF group and the control group, except for hyperlipidemia (*P* = 0.034) and calcium channel blockers status (*P* = 0.003), which were higher in the HF group.

### Serum Levels of miR-132 and miR-152

**Figure 1:**
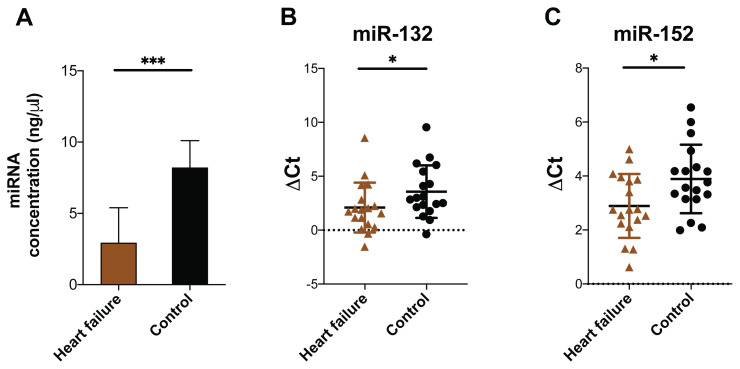
Levels of miRs in serum samples of HF and control groups. **A**. Total miR concentration (ng/μL) in serum samples of HF patients and controls determined by UV spectrophotometer. **B**. and **C**. ΔCt values of miR-132 and miR-152 from the serum samples of HF and control groups

We next quantified the levels of miR-132 and miR-152 in serum samples collected from HF patients and controls upon admission. The serum miRNA concentration of the control samples (8.21 ± 8.05 ng/μL) was significantly higher than that of the HF samples (2.93 ± 2.47 ng/μL; *P* = 0.0003) (Figure 1A). There was no difference in the mean Ct value of miR-132 (32.56 ± 3.38 versus 32.85 ± 4.02) and miR-152 (33.33 ± 3.38 versus 33.57 ± 2.01) between the HF and control groups, respectively. Following normalisation to the miR control, the mean ΔCt value of miR-132 was significantly lower in the HF group (2.09 ± 2.32) compared to that in the control group (3.57 ± 2.44; *P* = 0.029), indicating that there was a higher level of this miR in the serum samples of the HF patients (Figure 1B). A similar result was observed for the ΔCt value of miR-152 (2.89 ± 1.18 versus 3.89 ± 1.27; *P* = 0.044) (Figure 1C).

### Diagnostic Potential of miR-132 and miR-152 in Patients with Heart Failure

**Figure 2:**
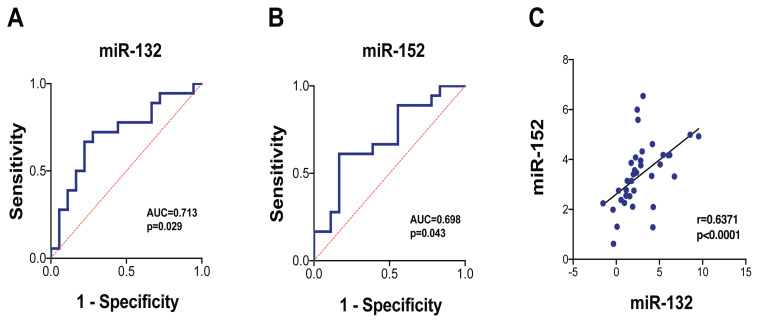
Discriminatory powers of serum miR-132 and miR-152. **A**. ROC curve for serum miR-132. **B**. ROC curve for serum miR-152. **C**. The correlation between miR-132 and miR-152

To assess whether serum miR-132 and miR-152 levels have potential diagnostic value in the identification of HF, we performed an ROC curve analysis for the ΔCt values of each selected miR. For miR-132, the area under the curve (AUC) value was 0.713 (95% CI: 0.54, 0.89; *P* = 0.029), with 77.8% specificity and 66.7% sensitivity (ΔCt cutoff value = 2.09) (Figure 2A). For miR-152, the AUC value was 0.698 (95% CI: 0.52, 0.87; *P* = 0.043), with 83.3% specificity and 61.1% sensitivity (ΔCt cutoff value = 2.96) (Figure 2B). A positive correlation was also observed between miR-132 and miR-152 (*r* = 0.6371, *P* < 0.0001) (Figure 2C).

## Discussion

In the present study, we measured the serum levels of miR-132 and miR-152 and assessed their diagnostic potential as candidate biomarkers for patients with HF. The total miR concentration was lower in the sera from the HF patients compared to that in the sera from the non-HF controls. In contrast, significantly higher levels of serum miR-132 and miR-152 were detected in the samples from the HF patients. The ROC curve analyses revealed that serum miR-132 and miR-152 each individually showed a moderate ability to discriminate HF patients from non-HF controls. In addition, a positive correlation was found between the serum levels of miR-132 and miR-152. To the best of our knowledge, this was the first study of circulating miRs in Vietnamese patients with HF; therefore, the data contribute to the establishment of miR panels associated with HF in this population.

A large body of research has indicated that circulating miRs are promising biomarkers for many heart diseases. Although they have been demonstrated to be highly stable under different storage conditions and unlikely affected by drug usage (18), changes in miR levels in cardiovascular patients have been observed. For example, an increase in serum miR concentration was reported in cases with myocardial infarction (33). However, in our study, lower serum miR concentrations were observed in the HF group compared to the control group. This finding aligns with that of another study in which lower miR levels were frequently detected in the blood of HF patients due to enhanced uptake of these molecules by cells to preserve cardiac functions (17). Since approximately 20 primary etiologies have been associated with HF (34), the underlying mechanism contributes to a reduction of serum miR levels in HF patients that may differ from myocardial infarction cases.

It has been shown that miR-132 plays a pivotal role in the regulation of multiple physiological processes of the heart (18) and its upregulation has not only been detected in the myocardium but also in the circulation of patients with HF. For example, Masson et al. (35) reported that plasma miR-132 was overexpressed in chronic HF cases and that higher levels were associated with more severe symptoms. In another study, which profiled circulating miR levels, it was confirmed that aberrant levels of miR-132 were present in HF patients with a reduced ejection fraction (36). Given that several groups have noted that there is disparate miR expression among sample types (serum, plasma or whole blood) (37, 38); our results suggested that miR-132 expression presented a consistent pattern between serum and plasma samples of HF patients, thereby favouring further research to evaluate the potential of miR-132 as a biomarker in larger cohorts of HF patients.

Previous functional studies have indicated that miR-152 mediates myocardial apoptosis and hypertrophy (39, 40) and that its dysregulation triggers the development and aggravation of HF through the Rho-kinase pathway (29). Even though miR-152 upregulation has been detected in failing human hearts (30), its circulating level has not been reported in HF patients up to now. An increase in plasma miR-152 has been associated with sudden cardiac death in cases with coronary syndrome (41), whereas ischaemic stroke patients were found to have a lower level of this miR in their sera (42). In our study, serum miR-152 overexpression was observed in HF patients. Together, these findings indicate that miR-152 may have divergent effects on, and/or be impacted in different ways by, cerebral and cardiovascular damage. Moreover, despite its potentially modest diagnostic value, serum miR-152 should be further assessed to clarify its clinical relevance in the context of HF.

There are certain limitations of this study that should be acknowledged. First, due to the very small number of participants, the results should not be over-emphasised and should only be considered as a foundation for the future evaluation of serum miR-132 and miR-152 as candidate biomarkers in studies with larger cohorts of HF patients. Second, miRNA levels were solely examined in samples collected on admission; thus, we cannot exclude the possibility that they were affected by the collection methods and timing. Hence, conducting a time-course analysis and taking repetitive measurements could provide more information about the dynamics of serum miR-132 and miR-152 expression and the potential applications of these molecules. Third, combination studies involving these miRs and other biomarkers are required to elucidate whether they have additive value in terms of the current methods used to diagnose HF. Furthermore, better stratification of patients may help to clarify possible associations between these miRs and particular HF etiologies.

## Conclusion

To the best of our knowledge, we have performed the first investigation to examine serum levels of miR-132 and miR-152 in patients with HF. Increased expression of these miRs was detected in the serum of patients with HF and the values could be used to distinguish HF patients from controls at a moderate level. Thus, miR-132 and miR-152 may be considered candidate biomarkers for HF diagnostic purposes. Further studies are required to identify the regulatory mechanism(s) underlying serum miR-132 and miR-152 upregulation and the dynamics of their expressions during HF processes. In addition, an expanded investigation of miRs associated with HF in the Vietnamese population is needed to determine their potential in enhancing the current diagnostic approaches used for this population.

## Figures and Tables

**Table 1:** Primers used for detecting miRs

Target gene	miRBase accession	Primer sequences
miR-132	MIMAT0000426	UAACAGUCUACAGCCAUGGUCG
miR-152	MIMAT0000438	UCAGUGCAUGACAGAACUUGG
miR-103a	MIMAT0000101	AGCAGCAUUGUACAGGGCUAUGA

**Table 2:** Characteristics of the study participants

Characteristics	Heart failure (*n* = 18)	Control (*n* = 18)	*P*-value
Gender, *n* (%)			1.000
Male	10 (55.6)	10 (55.6)	
Female	8 (44.4)	8 (44.4)	
Age (years old)^a^	58.7 (14.8)	59.2 (13.6)	0.862^b^
Body mass index (kg/m^2^)	23.9 (3.1)	23.9 (3.3)	0.967
Heart rate (beats/min)	83.6 (14.3)	76.8 (4.3)	0.164
Blood pressure (BP) (mmHg)			
Systolic BP	124.3 (18.1)	135.4 (19.6)	0.087
Diastolic BP^a^	75.9 (8.1)	75.9 (8.6)	0.948^b^
Cardiovascular risk factors, *n* (%)			
Hypertension	13 (72.2)	13 (72.2)	1.000
Diabetes	7 (38.9)	5 (27.8)	0.480
Hyperlipidemia	9 (50.0)	15 (83.3)	0.034*
History of myocardial infarction	7 (38.9)	5 (27.8)	0.480
Myocarditis	1 (5.6)	0 (0.0)	1.000^c^
Ischaemic heart diseases	6 (33.3)	4 (22.2)	0.457
Coronary syndrome	8 (44.4)	5 (27.8)	0.298
Angina pectoris	2 (11.1)	3 (16.7)	1.000^c^
Dilated cardiomyopathy	1 (5.6)	0 (0.0)	1.000^c^
Medications, *n* (%)			
Angiotensin receptor blocker	16 (88.9)	12 (66.7)	0.228^c^
Betablocker	17 (94.4)	14 (77.8)	0.338^c^
Statin	14 (77.8)	15 (83.3)	1.000^c^
Aspirin	5 (27.8)	2 (11.1)	0.402^c^
Clopidogrel	9 (50.0)	11 (61.1)	0.502
Calcium channel blocker	13 (72.2)	4 (22.2)	0.003*
Nitrate	0 (0.0)	1 (5.6)	1.000^c^

Notes:

* Statistical significance at *P*-value < 0.05;

a Median (IQR);

b Mann-Whitney U test;

c Fisher’s exact test
